# Transcriptomic dataset of the invasive species *Thunbergia alata* and its congener *Thunbergia grandiflora* (Acanthaceae) in Cali, Colombia

**DOI:** 10.1016/j.dib.2026.112861

**Published:** 2026-05-16

**Authors:** David Ruiz-Londoño, Pablo A. Pérez-Mesa, Harold Suárez-Baron

**Affiliations:** a Programa de Biología, Departamento de Ciencias Naturales y Matemáticas, Pontificia Universidad Javeriana Cali 760031, Colombia; b Semillero Eco-Evo-Devo, Facultad de Ingeniería y Ciencias, Pontificia Universidad Javeriana Cali 760031, Valle del Cauca, Colombia; c Max Planck Tandem Group, GEME, Facultad de Ciencias, Universidad Nacional de Colombia, Bogotá D.C. 111321, Colombia; d Institute for Bio- and Geosciences (IBG-4: Bioinformatics), CEPLAS, Forschungszentrum Jülich, Wilhelm Johnen Straße, Jülich 52425, Germany

**Keywords:** Adaptation, Differential gene expression, Functional annotation, Neotropics, Plant invasion, RNA-seq

## Abstract

This article presents the first comprehensive transcriptomic dataset of *Thunbergia alata* Bojer ex Sims, a fast-growing vine recognized as one of the most aggressive invasive plant species in the Neotropics. Native to eastern Africa and now widely naturalized across tropical and subtropical regions, *T. alata* exhibits remarkable ecological plasticity, allowing it to colonize diverse habitats and outcompete native vegetation. The availability of a complete transcriptomic resource for this species provides an essential foundation for exploring the molecular mechanisms underlying its invasiveness, rapid adaptation, and physiological resilience. By profiling transcriptomes from vegetative and reproductive tissues across multiple developmental stages in *Thunbergia alata*, this dataset opens new opportunities for comparative genomic and evolutionary studies aimed at understanding the genetic basis of invasion success in non-model tropical plants. Using RNA-seq, we generated *de novo* transcriptomes from vegetative and reproductive tissues at two developmental stages, complemented with comparative data from its non-invasive congener *Thunbergia grandiflora*. The dataset includes eight transcriptomes deposited in the National Center of Biotechnology Information (NCBI) Sequence Read Archive (SRA) database (SAMN51331451–SAMN51331458). Quality-filtered assemblies were evaluated with BUSCO, and differential expression analyses revealed thousands of genes potentially linked to adaptive processes, reproduction, stress responses, and invasiveness. These data provide a critical resource for studying the molecular mechanisms of invasiveness, plant adaptation, and comparative molecular evolution in non-model climbing species.

Specifications Table

**Table:** 

Subject	Biology
Specific subject area	Plant transcriptomics, invasive species biology
Type of data	Raw, processed, analyzed: raw reads (FASTQ), assembled transcriptomes (FASTA), expression matrices (counts, TPM), functional annotations.
Data collection	Vegetative (early; late) and reproductive (early; late) tissues from *T. alata*; vegetative, reproductive, and mixed tissues from *T. grandiflora*. Samples were collected in Colombia (Cali region), immediately frozen in liquid nitrogen; RNA extraction (TRIzol), Illumina NovaSeq 6000 sequencing (paired-end 150 bp), assemblies with Trinity, quality checks with FastQC/Trimmomatic, BUSCO completeness assessment.
Data source location	*Thunbergia alata:* La Elvira, Cali, Valle del Cauca, Colombia (N 3° 31′ 4.584′', W 76° 37′ 12.288′') Farallones de Cali, Valle del Cauca, Colombia (N 3° 19′ 52.385′', W 76° 38′ 9.808′') *Thunbergia grandiflora:* Pontificia Universidad Javeriana Cali, Colombia (N 3° 20′ 45″, W 76° 31′ 50″)
Data accessibility	Repository name: NCBI Sequence Read Archive (SRA) Data identification number: PRJNA1328341 BioSample: SAMN51331451 - SAMN51331458 Direct URL to data: https://www.ncbi.nlm.nih.gov/bioproject/PRJNA1328341
Related research article	None.

## 1 Value of the Data

• This study provides the first transcriptomic dataset of vegetative and reproductive tissues from *Thunbergia alata*, an invasive vine of global concern.
• The dataset enables comparative genomic and transcriptomic analyses between the invasive *T. alata* and the non-invasive *T. grandiflora* in Colombia.
• Supports studies in molecular ecology, conservation, and management of invasive species in the Neotropics.
• Can be used for evolutionary analyses, functional genomics, and development of molecular markers for monitoring invasions.

## 2 Background

**Fig. 1:**
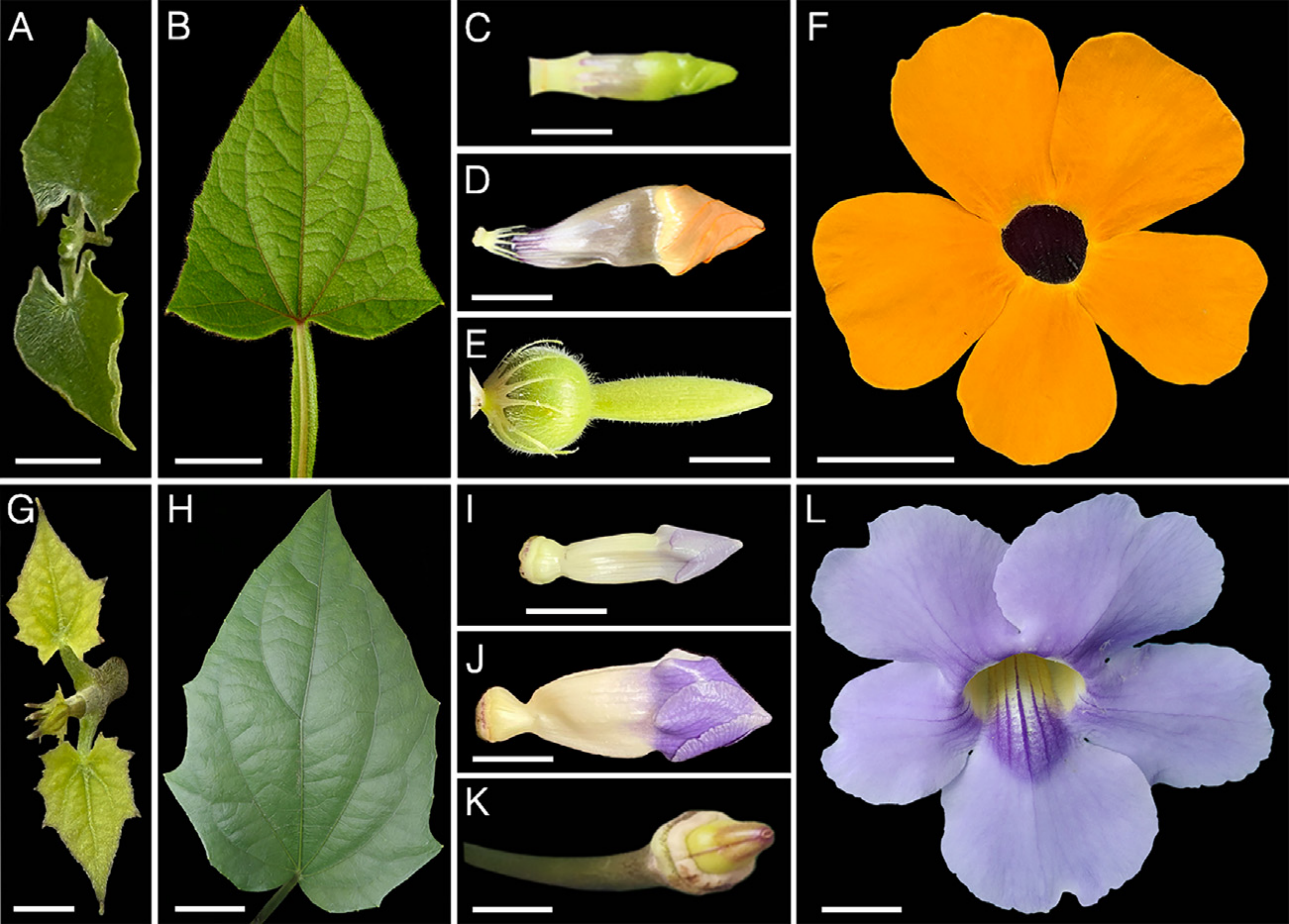
Vegetative and reproductive structures of *Thunbergia alata* and *Thunbergia grandiflora.* (A) Early vegetative stage of *T. alata* – young leaves (EV-TAL). (B) Late vegetative stage of *T. alata* – mature leaves (LV-TAL). (C) Early reproductive stage of *T. alata* – floral bud (ER-TAL). (D) Late reproductive stage of *T. alata* – preanthetic flower (LR-TAL). (E) Late reproductive stage of *T. alata* – fruit (LR-TAL). (F) Flower at anthesis, *T. alata.* (G) Early vegetative stage of *T. grandiflora* – young leaves, and (H) Late vegetative stage of *T. grandiflora* – mature leaves (V-TGR). (I) Early reproductive stage of *T. grandiflora* – floral bud, (J) Late reproductive stage of *T. grandiflora* – preanthetic flower, and (K) Late reproductive stage of *T. grandiflora* – fruit (R-TGR). (L) Flower at anthesis, *T. grandiflora.* Scale bars: (A-K) 1 cm; (H, L) 1.5 cm.

*Thunbergia alata* is an ornamental vine native to tropical Africa that has become naturalized and invasive in many tropical regions worldwide (Fig. 1A-F) [1]. Its vigorous growth, rapid vegetative propagation, and high adaptability to diverse environmental conditions have contributed to its success as an invasive species [2]. However, despite its ecological importance, the molecular mechanisms underlying its invasiveness remain poorly understood. To address this gap, we conducted a comparative transcriptomic analysis between *T. alata* and its congener *Thunbergia grandiflora* (Fig. 1G–L), two closely related climbers with contrasting invasive behaviors; while both species share similar climbing habits, *T. grandiflora* is less resilient to environmental change and does not exhibit the vegetative adaptive patterns observed in *T. alata* [3]. The generated RNA-seq data provide valuable genomic resources to identify biological processes associated with traits such as stress tolerance, growth regulation, and dispersal capacity, offering new insights into the genetic basis of plant invasiveness.

### 2.1 Objective

Invasive species threaten biodiversity and ecosystem function worldwide. *Thunbergia alata* is one of the most aggressive plant invaders in Andean ecosystems of Colombia and other tropical regions. However, little is known about its molecular mechanisms of adaptation. This dataset aims to provide publicly accessible transcriptomes that facilitate the discovery of the genetic basis of invasiveness and adaptative success, thereby supporting ecological genomics and conservation research.

## 3 Data Description

**Fig. 2:**
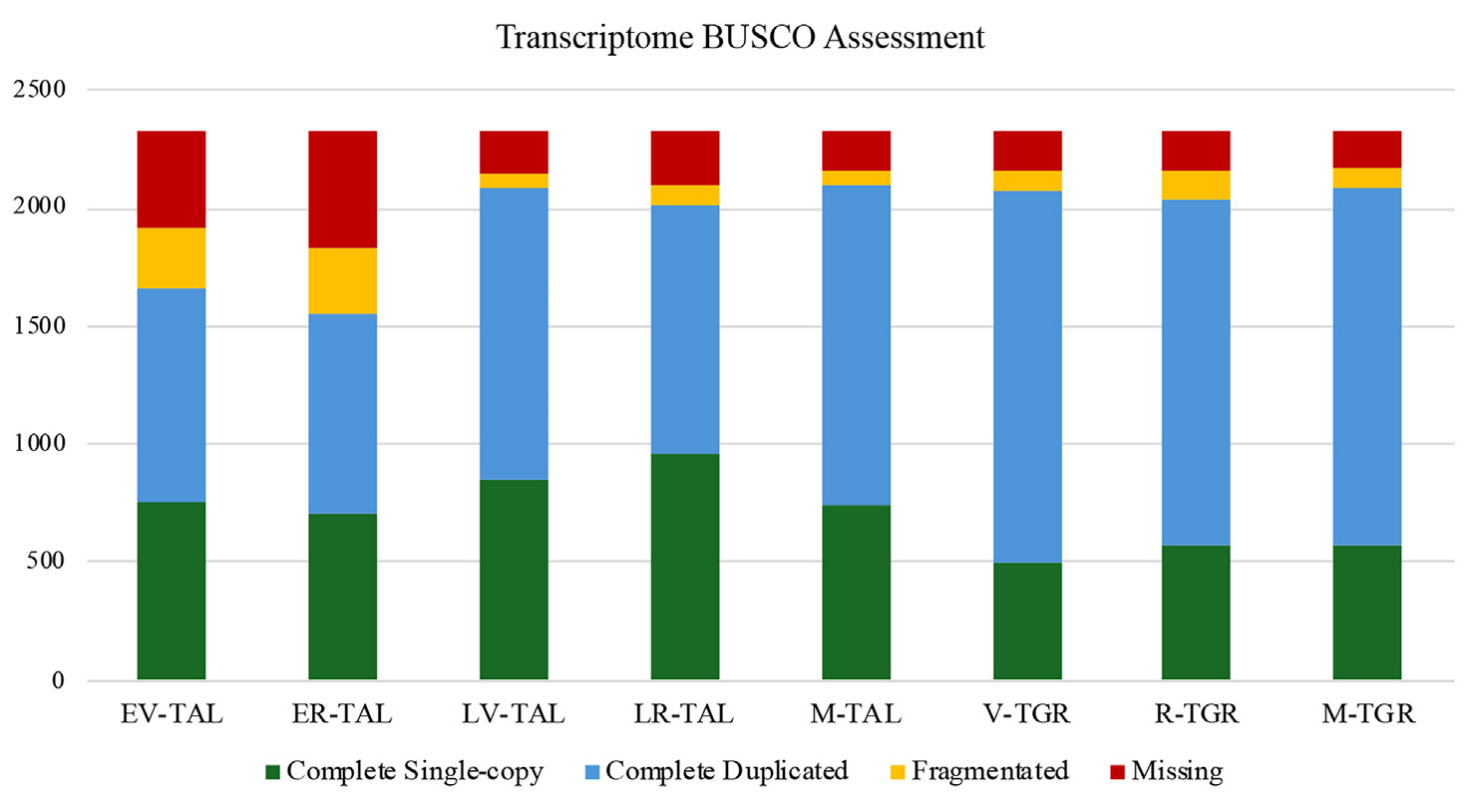
BUSCO assessment of transcriptome completeness across assemblies. The Y-axis shows the number of BUSCO ortholog groups mapped in the analysis; the X-axis lists each assembly. Blue represents the complete single-copy transcripts, green represents the complete duplicate copy genes, orange for fragmentated genes, and red for missing transcripts.

**Fig. 3:**
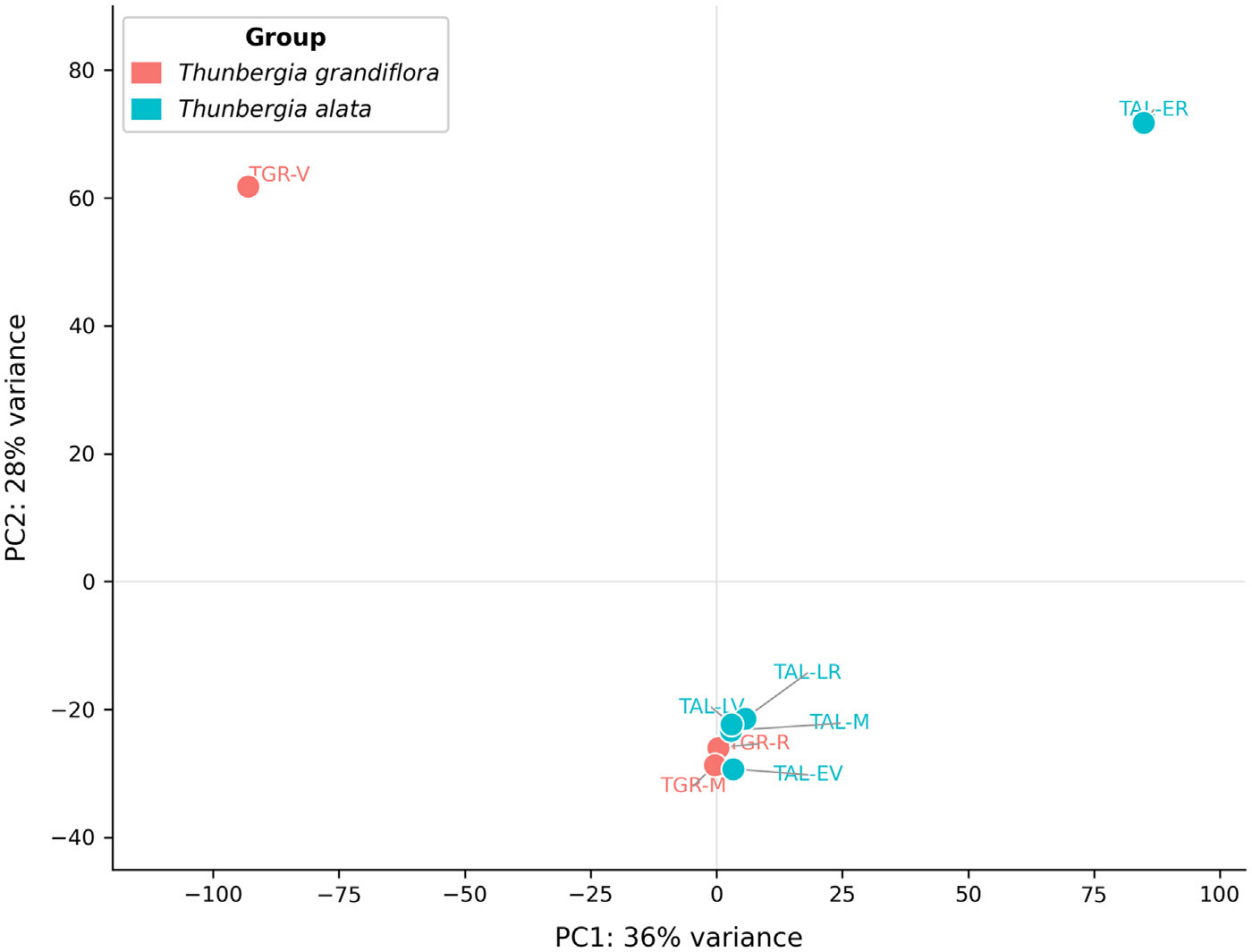
Principal component analysis (PCA) of VST-normalized gene expression counts across all eight transcriptome samples. Each point represents one sample colored by species: *Thunbergia grandiflora* (salmon) and *Thunbergia alata* (teal). Sample codes follow Table 1.

We generated a total of eight transcriptomes, including five from *Thunbergia alata* (EV-TAL, ER-TAL, LV-TAL, LR-TAL, and M-TAL) and three from *T. grandiflora* (V-TGR, R-TGR, and M-TGR) (Table 1 and Fig. 1). Samples M-TAL (Fig. 1A-E) and M-TGR (Fig. 1G-K) correspond to a mix of different reproductive and vegetative tissues at different developmental stages for each species. Each library produced between 29 and 51 million paired-end reads, with >94% of bases reaching Q30 quality (Table 1). *De novo* assemblies contained approximately 100,000 to 190,000 transcripts per library, with N50 values ranging from 1054 to 1714 bp (Table 2). BUSCO v5.8.0 [6] assessments against the eudicots_odb10 lineage set indicated high assembly completeness, with >85% of conserved orthologs recovered across all assemblies (Fig. 2). Differential expression analyses revealed thousands of differentially expressed genes (DEGs) both within *T. alata* (comparing early vs. late stages and vegetative vs. reproductive tissues) and between *T. alata* and *T. grandiflora*. Candidate genes included those associated with pollen and fruit development, trichome morphogenesis, stress tolerance, and acclimation processes. All BioSamples and transcriptomes are publicly available through the GenBank BioSample database under accessions SAMN51331451–SAMN51331458. A principal component analysis (PCA) of VST-normalized gene expression counts is shown in Fig. 3.

**Table 1:** RNA sequencing output and quality statistics for five tissue samples of *Thunbergia alata* (TAL) and three tissue samples of *Thunbergia grandiflora* (TGR), including total raw and clean reads, sequencing yield, and base quality scores (Q30).

Sample	Total Raw Reads	Total Clean Reads	Total Bases (Gb)	Clean Reads Ratio (%)	Q30 (%)
EV-TAL	21,674,025	21,674,022	3.2	99.99	94.24
ER-TAL	24,728,010	24,728,010	3.6	100	94.26
LV-TAL	14,794,388	14,794,387	2.2	99.99	94.64
LR-TAL	19,643,930	19,643,929	2.9	99.99	94.82
M-TAL	25,699,449	25,699,444	3.8	99.99	94.54
V-TGR	19,192,406	19,192,404	2.8	99.99	94.61
R-TGR	19,372,566	19,372,561	2.8	99.99	94.78
M-TGR	20,483,087	20,483,082	3.0	99.99	94.54

**Table 2:** *De novo* transcriptome assembly metrics for five tissue samples of *Thunbergia alata* (TAL) and three tissue samples of *Thunbergia grandiflora* (TGR), including contig number, total length, mean length, N50, and GC content.

Sample	Total Number	Total Length (bp)	Mean Length	Contig N50	GC (%)
EV-TAL	102,399	85,646,711	611	1280	42.32
ER-TAL	163,253	107,435,389	390	1054	43.36
LV-TAL	106,883	116,273,840	743	1714	41.42
LR-TAL	106,077	113,695,793	732	1694	41.78
M-TAL	160,996	140,439,994	501	1493	42.12
V-TGR	185,520	170,256,254	637	1383	41.47
R-TGR	184,345	165,197,257	639	1331	41.64
M-TGR	192,697	173,831,893	626	1383	41.47

Keys:.

EV-TAL: Early vegetative *T. alata*.

ER-TAL: Early reproductive *T. alata*.

LV-TAL: Late vegetative *T. alata*.

LR-TAL: Late reproductive *T. alata*.

M-TAL: Mix *T. alata*.

V-TGR: Vegetative *T. grandiflora*.

R-TGR: Reproductive *T. grandiflora*.

M-TGR: Mix *T. Grandiflora*.

## 4 Experimental Design, Materials and Methods

### 4.1 Plant material

Samples of *Thunbergia alata* were collected from two naturalized populations in Cali, Colombia (La Elvira and Farallones de Cali), while *T. grandiflora* samples were obtained from individuals established on the campus of Pontificia Universidad Javeriana Cali. For *T. alata*, five tissue/stage combinations were sampled: early vegetative, late vegetative, early reproductive, late reproductive, and a mixed sample. For *T. grandiflora*, sampling did not resolve developmental stages, and tissues were grouped into three categories: vegetative, reproductive, and mixed. Samples were frozen in liquid nitrogen and stored at −80 °C until extraction.

### 4.2 RNA extraction and sequencing

Total RNA was extracted using TRIzol reagent (Invitrogen, Carlsbad, CA, USA) following the manufacturer's protocol. RNA integrity and concentration were assessed with a NanoDrop spectrophotometer (Thermo Fisher Scientific, Waltham, MA, USA) and an Agilent Bioanalyzer (Agilent Technologies, Santa Clara, CA, USA); only samples with RIN > 7 were retained for library preparation. Low-input eukaryotic mRNA libraries were constructed and sequenced by Novogene (Sacramento, CA, USA) on an Illumina NovaSeq 6000 platform (Illumina, San Diego, CA, USA) using paired-end 150 bp (PE150) reads.

### 4.3 *De novo* transcriptome assembly

Raw read quality was evaluated with FastQC (v0.12.1) and adapter trimming and quality filtering were performed with Trimmomatic (v0.36) [4]. The resulting clean reads were used for *de novo* transcriptome assembly with Trinity (v2.15.1) [5], and assembly completeness was assessed against the eudicots_odb10 database using BUSCO (v5.8.0) [6].

### 4.4 Differential expression analysis

Transcript abundance was estimated by mapping reads with Kallisto (v0.48.0) [7], and differentially expressed genes (DEGs) were identified with DESeq2 (v1.40.2) [8] applying a false discovery rate threshold of ≤ 0.05 and an absolute fold change > 5.

### 4.5 Functional annotation

Functional annotation was carried out using TRAPID (v2.0) [9] and the Blast2GO [10] module in OmicsBox (v3.4) for Gene Ontology (GO) classification, complemented by a targeted search for genes associated with invasiveness, including *HSP70, EIN3*, and *JAR1*.

## Limitations

Sample replicates were pooled and did not sequence separately. However, the dataset remains robust for exploratory and comparative transcriptomic analyses.

## Ethics Statement

In this work no protected or endangered species were sampled. All authors of the aforementioned scientific data reporting publication acknowledge and affirm that they have thoroughly reviewed and complied with the ethical guidelines for publication in Data in Brief. Furthermore, they confirm that the present study does not involve the participation of human subjects, animal experimentation, or the collection of data from social media platforms.

## CRediT Author Statement

David Ruiz-Londoño: Methodology, data curation, analysis, writing – original draft & editing; Pablo A. Pérez-Mesa: Conceptualization, supervision, writing – review & editing; Harold Suárez-Baron: Conceptualization, project administration, funding acquisition, writing – review & editing.

## Data Availability

NCBI BioSample accessions:

*Thunbergia alata*:

SAMN51331451

SAMN51331452

SAMN51331453

SAMN51331454

SAMN51331455

*Thunbergia grandiflora*:

SAMN51331456

SAMN51331457

SAMN51331458

Thunbergia invasiveness (Original data) (SRA).
